# Biased perceptions explain collective action deadlocks and suggest new mechanisms to prompt cooperation

**DOI:** 10.1016/j.isci.2021.102375

**Published:** 2021-03-29

**Authors:** Fernando P. Santos, Simon A. Levin, Vítor V. Vasconcelos

**Affiliations:** 1 Department of Ecology and Evolutionary Biology, Princeton University, Princeton, NJ 08544, USA; 2 Center for BioComplexity, High Meadows Environmental Institute, Princeton University, Princeton, NJ 08544, USA; 3 Informatics Institute, University of Amsterdam, Science Park 904, 1098XH Amsterdam, The Netherlands; 4 Resources for the Future, Washington, DC, USA; 5 Beijer Institute of Ecological Economics, Stockholm, Sweden; 6 Andlinger Center for Energy and the Environment, Princeton University, Princeton, NJ 08544, USA; 7 Institute for Advanced Study, University of Amsterdam, 1012 GC Amsterdam, The Netherlands; 8 Centre for Urban Mental Health, University of Amsterdam, Amsterdam, The Netherlands; 9 Princeton Institute for International and Regional Studies, Princeton University, Princeton, NJ 08544, USA

**Keywords:** Psychology, Sociology, Decision Science

## Abstract

When individuals face collective action problems, their expectations about others' willingness to contribute affect their motivation to cooperate. Individuals, however, often misperceive the cooperation levels in a population. In the context of climate action, people underestimate the pro-climate positions of others. Designing incentives to enable cooperation and a sustainable future must thereby consider how social perception biases affect collective action. We propose a theoretical model and investigate the effect of social perception bias in non-linear public goods games. We show that different types of bias play a distinct role in cooperation dynamics. False uniqueness (underestimating own views) and false consensus (overestimating own views) both explain why communities get locked in suboptimal states. Such dynamics also impact the effectiveness of typical monetary incentives, such as fees. Our work contributes to understanding how targeting biases, e.g., by changing the information available to individuals, can comprise a fundamental mechanism to prompt collective action.

## Highlights

• Individuals often misperceive the real cooperation levels in a population
• We model the impact of such biases in non-linear public goods games dynamics
• False uniqueness and false consensus can lock groups in suboptimal states
• Addressing perception biases can be more effective than typical monetary incentives

## Graphical abstract

**Figure:**
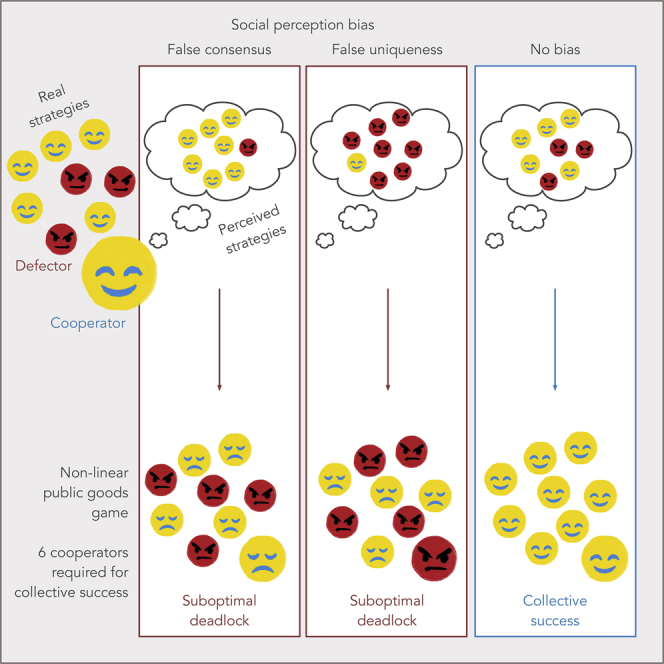

## Introduction

Many of the most pressing problems humanity faces today share the perils of public goods dilemmas (Dietz et al., 2003; Olson, 1965). These are dilemmas in which reaching a minimum level of cooperation is necessary to achieve the best social outcome, but in which refusing to do so (free-riding) is the immediate rational action to follow. Greenhouse gas emissions, overexploitation of natural resources, low vaccination coverage, antibiotics abuse, or fertilizer overuse are challenges in which incentivizing cooperation is arduous yet necessary to obtain results that benefit all (Dietz et al., 2003; Keohane and Victor, 2016; Levin, 1999; Smith et al., 2005). Failing to do so leads to the infamous *tragedy of the commons* (Hardin, 1968), engendering ecological breakdown and increased inequality, resource depletion, failure to achieve herd immunity, antimicrobial resistance, or groundwater contamination. Averting those scenarios requires judiciously designing incentives, interventions, and institutions.

Cooperation in public goods games is constrained not only by the costs and benefits involved but also by the social environment wherein the interactions take place. Experiments in the laboratory (Fischbacher et al., 2001) and the field (Frey and Meier, 2004) reveal that “those who believe others will cooperate in social dilemmas are more likely to cooperate themselves (Ostrom, 2000).” Elinor Ostrom identifies this as one of the seven stylized facts about public goods games—results replicated so frequently that they can be considered core facts*.* In fact, this finding has accompanied public goods games since the very first experiments with this interaction paradigm, which already indicate that assumptions about others' behavior impact the decision to cooperate (Dawes et al., 1977). Recent research reinforces this idea, revealing that second-order beliefs (i.e., beliefs about others' beliefs) are good predictors of one's own behavior (Jachimowicz et al., 2018). This observation underscores the potential effectiveness of norm-based interventions whereby informing individuals about the cooperative actions of others constitutes a trigger for cooperation (Bicchieri, 2016; Carattini et al., 2019; Miller and Prentice, 2016; Nyborg et al., 2016).

Although there is a link between cooperation and beliefs about others cooperating, humans reveal social perception biases, e.g., systematic errors in estimating the distribution of cooperative behaviors in a population. In a paradigmatic example, Monin and Norton report that, in a field study during a water shortage crisis in which students were asked to reduce the number of showers to save water, individuals systematically failed to estimate the prosocial behavior of others (Monin and Norton, 2003). Limiting water usage (reducing the number of showers) has all the ingredients of cooperation, whereas refusing to do so implies defecting on the public good. Survey results show that students concurred in false consensus, uniqueness bias, pluralistic ignorance, and other typical social perception biases. Beyond local public goods, the existence of perception bias extends to climate change beliefs. Research has shown that both the mass public and political elites—in China, the United States, and Germany—tend to underestimate the pro-climate positions of others (Mildenberger and Tingley, 2019; Taddicken et al., 2019). Likewise, Leviston et al. investigate the existence of pluralistic ignorance and false consensus effects regarding climate change beliefs in Australia, finding that opinions are subject to strong false consensus; in general, people underestimate the number of others who agree with the existence of climate change (Leviston et al., 2013). Although those opinions do not directly translate into cooperation or defection behaviors, they can be thought of as a proxy for engaging (or not) in climate action. The existence of such social perception biases was recently pointed out as an impediment to discussions about climate change (Geiger and Swim, 2016)—leading to the so-called spiral of silence (Noelle-Neumann, 1974)—being one possible reason for inhibition to take part in collective climate action (Kjeldahl and Hendricks, 2018). All the biases mentioned have for long been known in social psychology: Pluralistic ignorance is known as a situation in which people erroneously believe that their private opinions or behaviors are different from everybody else's (Miller and McFarland, 1987; Prentice and Miller, 1993)—which corresponds to false uniqueness or uniqueness bias when actions map with personal injunctive norms (Goethals et al., 1991; Suls and Wan, 1987); False consensus is known as the tendency to overestimate the representativeness of one's opinion or behavior in a population (Ross et al., 1977). Given the above-mentioned connection between cooperation in public goods dilemmas and beliefs about others' cooperative behavior, it is likely that such biases play an influential role in collective action itself.

The effect of perception biases is likely to be exacerbated in non-linear public goods games, in which collective action cannot be decomposed into pairwise interactions. A prototypical example is that of threshold public goods games, where the benefits of cooperation are not realizable until a certain fraction of cooperators exists (e.g., the advantages of reducing carbon emissions only ensue once a certain fraction of countries or industries do so) (Milinski et al., 2008; Pacheco et al., 2009; Santos and Pacheco, 2011; Tavoni et al., 2011). Threshold formulations for interactions typically lead to tipping points, characteristic of social behavior influenced by social norm change and expected to play a critical role in transitions to sustainability, e.g., mass adoption of sustainable technologies, implementation of collective insurance and risk-mitigation strategies (Santos et al., 2021), or changes in diets (Nyborg et al., 2016). Cooperation might, in this case, be hampered by failing to estimate accurately the number of individuals willing to cooperate, either by overestimating their real number (“*there are so many cooperators, I do not need to cooperate”*) or underestimating it (“*there are too few cooperators, it is not worth it for me to cooperate”*). Likewise, biases may create the illusion that the required number of cooperators is closer to the goal than it is, thus motivating cooperation. Importantly, these (incorrect) expectations about others can persist even after repeated interactions (Ackermann and Murphy, 2019). Therefore, it is fundamental to (1) understand the role of social perception bias in the dynamics of (non-linear) public goods games and (2) understand how to design cooperation incentives and interventions in situations where perception bias is prevalent.

**Figure 1:**
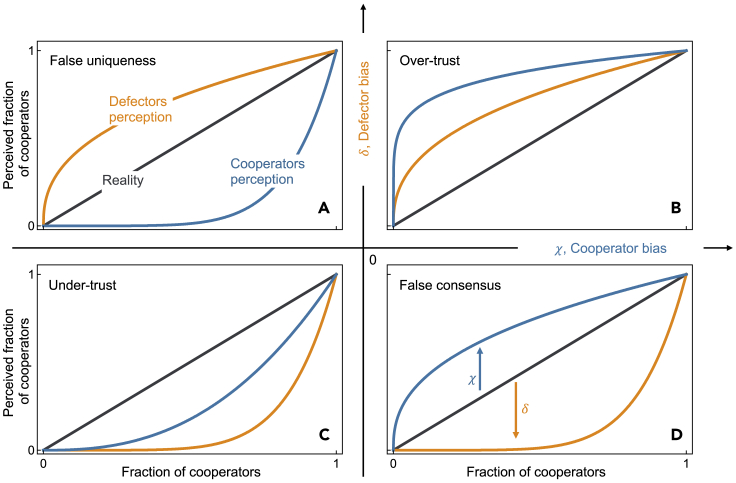
Individual perception biases toward cooperation Individuals can be affected by different biases, depending on their behavior. Cooperators can perceive a higher or lower fraction of cooperators than in reality, and so do defectors. This creates the four different scenarios represented. (A) “False uniqueness” corresponds to a case in which both cooperators and defectors believe their representation in the population is a smaller fraction than it is. (B) “Over-trust” reflects biases where all individuals believe there are more cooperators than there are. (C) “Under-trust” reflects a belief that there are fewer cooperators than there are. (D) In the “false consensus” scenario, cooperators and defectors believe their representation is broader than it is.

We provide a theoretical model to analyze the effect of perception bias in public goods cooperation dynamics. We consider a population of (boundedly) rational individuals who adapt their behavior through a (smooth) best response (Fudenberg et al., 1998) while possibly incurring perception bias—either under- or overestimating the overall levels of cooperation. As detailed below (see transparent methods, supplemental information), we assume a population in which each individual can either adopt strategy C (cooperate) or D (defect). Interacting groups are formed randomly. Each cooperator pays a cost *c* > 0, and, when there are more than a threshold number, *M*, of cooperators, everyone gets a benefit, *b* > *c*, plus an enhanced share of the contributions of cooperators. We focus the analysis in situations where the enhancement, *f*, is such that there is an individual incentive to cooperate above the threshold (*f* > 1), and both full cooperation and full defection are Nash equilibria—with full cooperation being the social optimum. Above the threshold, cooperation is self-enforceable (Keohane and Victor, 2016), yet it is potentially hard to trigger in the first place, when below the threshold. This regime allows us to focus on the simpler situation in which collective action dynamics, in the absence of bias, are characterized by a single coordination barrier (see the supplemental information for further exploration of the parameters, where we show that the effects of biases discussed in the main text extend to other types of collective action dilemmas). For a given configuration of the population, individuals will adapt by selecting the strategy maximizing their payoffs, given an estimate of the current distribution of strategies. Such estimates can be biased. As Figure 1 conveys, all the perception biases we consider here can be situated in a two-dimensional space (*χ*,*δ*), defined by a bias in the level of cooperators by cooperators (*χ*, where *χ* < 0 implies an underestimation and *χ* > 0 an overestimation of the number of other cooperators in the population) and a bias in the level of cooperators by defectors (*δ*, where *δ* < 0 implies an underestimation and *δ* > 0 an overestimation in the number of cooperators by defectors). Within this space, we can identify four distinct types of social perception biases: (1) “False uniqueness” (*δ* > 0, *χ* < 0), in which both cooperators and defectors believe their representation in the population is a smaller fraction than it is (we include a note on this definition of false uniqueness in supplemental information); (2) “Over-trust” (*δ* > 0, *χ* > 0), which reflects biases where all individuals believe there are more cooperators than there is; (3) “Under-trust” (*δ* < 0, *χ* < 0), which reflects a belief that there is less cooperation than there is; and (4) “False consensus’” (*δ* < 0, *χ* > 0), whereby both cooperators and defectors believe their representation is broader than it is.

## Results

The aforementioned biases have substantial impacts on the dynamics of cooperation. We first focus on the role of homodirectional biases, affecting cooperators and defectors alike (over-trust and under-trust, Figures 1B and 1C), and then move to heterodirectional biases, which affect cooperators and defectors in opposite ways (false uniqueness and false consensus, Figures 1A and 1D).

### Under-trust and over-trust impact the likelihood to reach optimal coordination

**Figure 2:**
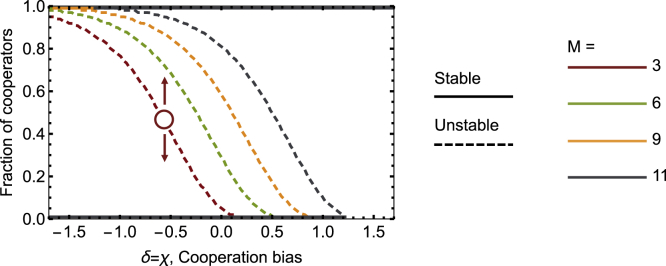
Under-trust and over-trust (homodirectional biases) impact the likelihood of reaching optimal coordination In a coordination dilemma (*f* > 1), when there is no bias (δ = χ = 0), the dynamics of the population are characterized by a coordination threshold that corresponds to the fraction of cooperators above which the population will evolve toward full cooperation and below which it will evolve toward defection. That coordination threshold depends on the threshold within the interacting group, М, necessary for getting the reward. The dashed lines represent unstable equilibria: below them, there are insufficient cooperators, and the population evolves to a state of full defection; above, the population evolves to a state of full cooperation. Full lines at 0 and 1 represent stable equilibria in the fraction of cooperators. The left side of the figure, with negative biases toward cooperation (*δ* = *χ* < 0), is part of the under-trust region. The right side, with positive biases toward cooperation (*δ* = *χ* > 0), is part of the over-trust region. Over-trust promotes the coordination of a population into a cooperative state, whereas under-trust does the opposite. Effectively, biases toward the existence of cooperators reduce the coordination threshold, facilitating cooperation. Parameters: *N* = 11, *c* = 1, *b* = 10, *f* = 1.5, and *χ* = *δ*.

In the game considered here, and detailed above, collective benefits are distributed—and cooperation becomes desirable both for the group and the individuals—when a minimum fraction of cooperators exist in a population. In Figure 2, we control δ and χ such that we navigate from a scenario of under-trust (Figure 1C) into a scenario of over-trust (Figure 1B). We can observe that increasing cooperation bias (i.e., increasing both δ and χ) eases the coordination toward full cooperation entailed by the non-linear public goods with *f* > 1. If individuals mistakenly perceive that there are more cooperators in a population than there truly are, they may recognize that the collective benefits of cooperation can be attained, even in a configuration where the number of cooperators is still insufficient. Conversely, reducing cooperation bias (i.e., decreasing both δ and χ) induces individuals to understand that the collective benefits of cooperation are harder to be reached, even in situations where, actually, there are a sufficient number of cooperators to realize collective success. As such, under-trust hinders coordination toward full cooperation, requiring a higher number of cooperators to have a population self-organize toward the socially desirable outcomes. The effect of over- and under-trust on coordination toward cooperation can be grasped by the position of the coordination point in Figure 2: for different values of *M*, increasing *δ* and *χ* reduces the position of the coordination point (represented with dashed lines), implying that a smaller fraction of cooperators is needed to evolve toward full cooperation.

### False uniqueness and false consensus lead to suboptimal deadlocks

**Figure 3:**
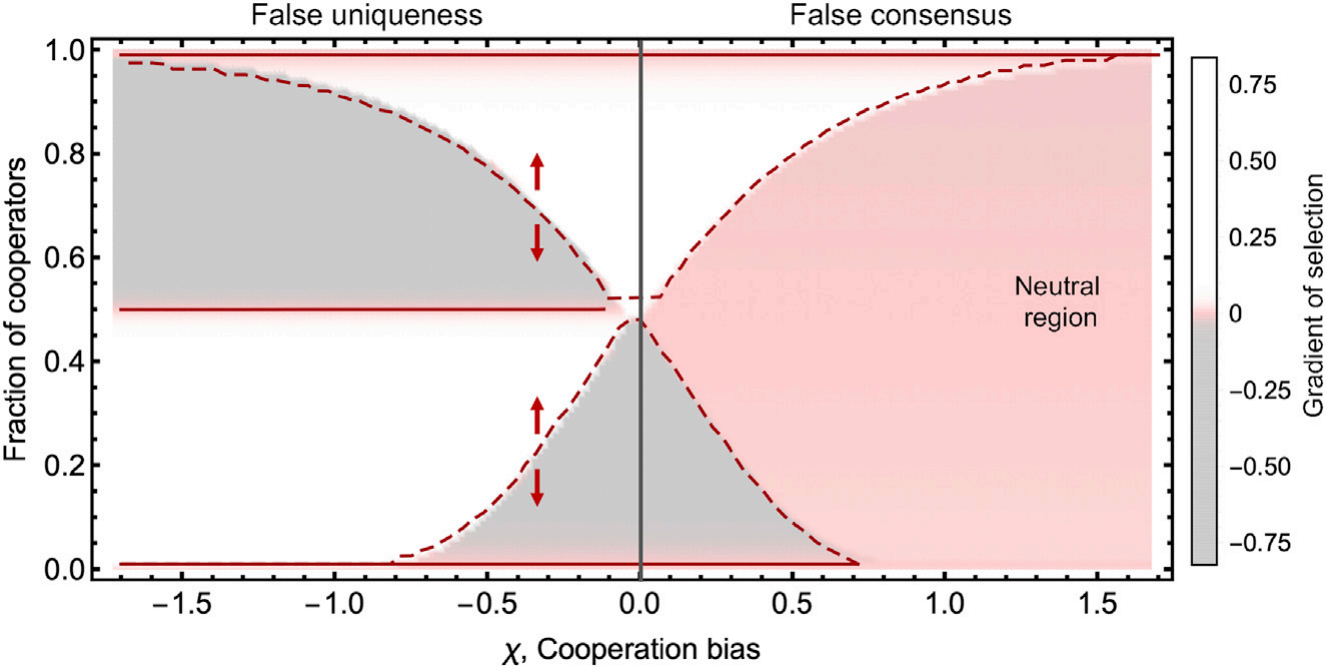
False uniqueness and false consensus (heterodirectional biases) lead to deadlocks resulting in individual and collective suboptimal configurations We show the position of the equilibrium points associated with different biases. Dashed lines represent unstable equilibria, and full lines represent stable equilibria. Positive (negative) values of the gradient of selection, in white (gray), indicate a tendency for the number of cooperators to increase (decrease). False uniqueness (*χ* < 0, left) is characterized by the existence of a stable configuration in which cooperators and defectors coexist, and the population is unable to solve the coordination dilemma. From the social-optimum point of view, this is the worst-case scenario because individuals contribute but not enough to surpass the threshold. A second—higher—coordination needs to be achieved for the population to reach a fully cooperative state. False consensus introduces a region where individuals believe there are no incentives to changing strategy even though the population is in a suboptimal configuration from the individual and collective point of view. In such a region, individuals do not change strategies, and the gradient of selection is 0 (neutral region, pink). Again, a second, higher, coordination needs to be achieved for the population to reach a fully cooperative state. Same parameters as Figure 1, with *M* = 8 and *δ* = −*χ*.

The effects observed in Figure 2 result from homodirectional bias, that is, situations in which both cooperators and defectors over- or underestimate the real number of cooperators in a population. Social perception bias can, however, affect cooperators or defectors in different directions. In the case of false consensus (Figure 1D), individuals overestimate the adoption of their own strategy in a population, meaning that cooperators will overestimate the fraction of cooperators and defectors will overestimate the fraction of defectors. If one considers heterodirectional biases of this kind, the effects on cooperation dynamics become more intricate. Figure 3 summarizes the effects of heterodirectional bias on cooperation dynamics, considering false uniqueness (*δ* = −*χ*, *χ* < 0, left half of the figure) and false consensus (*δ* = −*χ*, *χ* > 0, right half). We can observe that false uniqueness induces a stable coexistence of cooperators and defectors, which may not be sufficient to support high levels of collective success (see transparent methods, supplemental information, for more details on group achievement). On the other hand, false consensus introduces a “neutral region” in which both cooperators and defectors stick to their current strategy.

**Figure 4:**
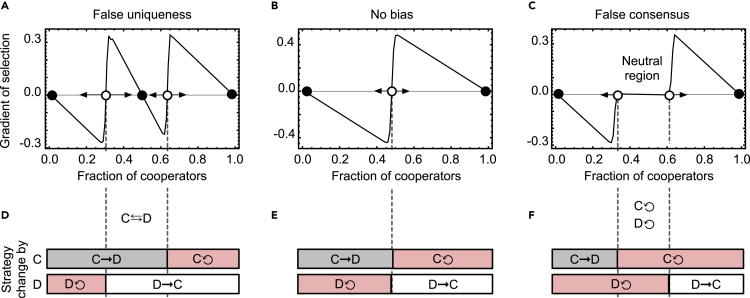
False uniqueness originates a stable cooperator-defector coexistence, whereas false consensus introduces a neutral region on cooperation dynamics (A–F) The gradient of selection (A–C) measures how likely it is for cooperators to spread in a population, compared with defectors. Positive gradient values mean that cooperators are more likely to spread than defectors. As noted in Figure 3, false uniqueness induces a stable coexistence of cooperators and defectors (A). Further inspection of the strategic dynamics informs that this coexistence is due to a recurring transition of cooperators into defectors and defectors into cooperators (D). Given that individuals adopting a given strategy underestimate the representativeness of that behavior, everyone is inclined to change strategies: cooperators, as they do not believe that a minimal threshold of cooperation can be reached; defectors, as they believe that the threshold was already reached. For reference, we include the gradient corresponding to the no-bias situation (B and E); in that case, stabilizing cooperation requires overcoming one coordination barrier. If false consensus prevails, we note an inactivity area (neutral region, C) where both cooperators and defectors are satisfied with their strategy. Individuals overestimate the representativeness of their strategy in the population; as such, cooperators keep their strategy as they believe that the cooperation threshold was already reached, whereas defectors keep defecting as they believe that the threshold can never be reached (F). We consider *χ* = −*δ* = −0.2 (false uniqueness, A) *χ* = *δ* = 0 (no bias, B), and *χ* = − *δ* = 0.2 (false consensus, C). Same parameters as in Figure 2. See also Figure S1 for analysis of the effects of spontaneous changes and errors.

The different impacts of false consensus and false uniqueness on cooperation dynamics can be further understood if we examine the gradients of selection and the decisions characterizing each type of bias. Figure 4B shows the original selection gradient in the absence of any bias. As already discussed, in this case, the dynamics are simply characterized by a coordination threshold that corresponds to the fraction of cooperators above which the population will evolve toward full cooperation and below which it will evolve toward defection. As Figure 4E reveals, below that threshold, cooperators turn into defectors with high probability and defectors remain defectors, making the gradient of selection of cooperators negative. Above that threshold, defectors are likely to turn into cooperators, and cooperators stick to their strategy, making the gradient of selection of cooperators positive. If individuals undergo false uniqueness biases (Figure 4A), we observe that, at the macroscopic level, the population is likely to remain in a state where cooperators and defectors coexist. In Figure 4D, we can observe that this coexistence is motivated by a set of configurations in which both cooperators and defectors are likely to change their strategies: cooperators believe themselves to be surrounded by defectors, which motivates them to alter their strategy to defection; conversely, defectors expect that more cooperation exists than what actually occurs, which encourages themselves to become cooperators. A different dynamic is sustained by false consensus (Figure 4C). In this case, we observe an area in which any change in behaviors only occurs through exogenous factors (see supplemental Information). By further inspecting the likelihood that individuals change their strategy (Figure 4F), we realize that a neutral region appears when neither cooperators nor defectors are incentivized to alter their strategies: as everyone overestimates the representativeness of their own strategy in the population, cooperators believe that the cooperation threshold will be achieved, thus expecting to receive high benefits for cooperating, and defectors are convinced that such threshold is hardly attained, assuming no benefits for starting cooperating.

**Figure 5:**
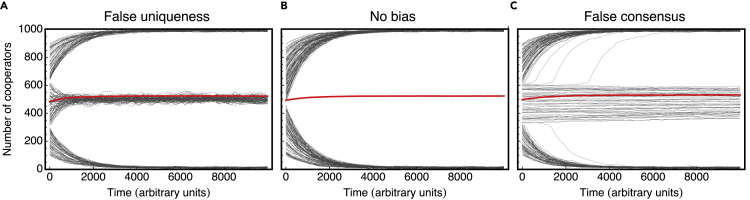
False uniqueness originates a stable cooperator-defector coexistence, whereas false consensus introduces a neutral region on cooperation dynamics (time-series analysis) (A–C) We simulate the time evolution of strategy adoption in large populations composed of (*Z* = 1,000) individuals incurring (A) false uniqueness, (B) no bias, or (C) false consensus. Each gray curve corresponds to a single run starting from a random initial condition (in terms of the initial number of cooperators). The red curve corresponds to the average over all runs. We confirm that false uniqueness originates a prevalent cooperator-defector coexistence, where populations with an intermediate number of initial cooperators get trapped in a deadlock configuration. False consensus, on the other hand, leads to a neutral region where individuals maintain their strategies (eventually approaching the limits of such area and evolving to either full cooperation or full defection). Same parameters as in Figure 2.

The previous results are confirmed in Figure 5 by a time-series analysis, where we assume that a large population of individuals (*Z* = 1,000) evolve following the best-response process detailed above (and in the transparent methods section, supplemental information). We confirm that false uniqueness originates a prevalent cooperator-defector coexistence, and false consensus introduces a neutral region where, over time, individuals maintain their strategies; both scenarios are sub-optimal, leading to many groups failing to achieve collective success.

### Perception biases affect the effectiveness of monetary incentives

The previous effects of perception bias on cooperation dynamics imply that (1) different biases may have an impact on achieving high levels of collective success and (2) interventions are likely to have a different impact depending on whether individuals in a given population reveal a specific bias. Reasoning about bias and incentives simultaneously also suggests comparing the effect of interventions based on (possibly monetary) incentives such as rewards and punishment (Andreoni et al., 2003; Balliet et al., 2011; Couto et al., 2020; Dreber et al., 2008; Góis et al., 2019; Vasconcelos et al., 2013) with the effect of interventions that alter the information landscape available to individuals, akin to norm-based interventions (Carattini et al., 2019; Miller and Prentice, 2016; Nyborg et al., 2016). Monetary incentives and information (media) campaigns are typical tools to change norms and behaviors (Bicchieri, 2016). We should also note that individuals tend to overestimate the impact of self-interest on the attitudes and behaviors of others (Miller and Ratner, 1998), and this tendency is particularly salient when information is incomplete (Vuolevi and Van Lange, 2010), which again denotes an interplay between monetary incentives (appealing to self-interest) and information incentives (attempting to reduce uncertainty).

**Figure 6:**
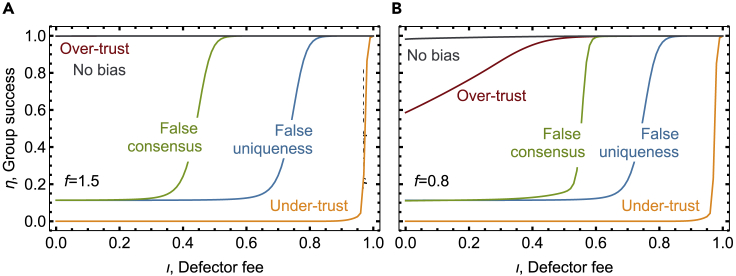
Perception biases affect the effectiveness of monetary incentives (such as a fee to be paid by defectors) Incentives, like reward or punishment, are often used to move populations from unfavorable to favorable equilibria. The effectiveness of incentives, however, depends on the level and nature of biases existent in a population. Here, we measure group success, i.e., the fraction of groups that, on average, have the necessary number of cooperators to reap the benefits of collective action. We explore a game setting in which unbiased individuals self-organize toward high levels of group success. In a population with individuals that over-trust (*χ* = *δ* = 0.6), extra incentives are unnecessary to achieve group success if full cooperation is an equilibrium (panel A, *f* = 1.5); incentives also improve cooperation when individuals over-trust and if there is no incentive for cooperation above the threshold of group success (panel B, *f* = 0.8). In this case, over-trusting individuals may refrain from cooperating when they erroneously believe that the collective success threshold was already achieved. If individuals incur in false consensus (*χ* = −*δ* = 0.6), a lower punishment on defectors (or conversely, reward to cooperators) is necessary, compared with a scenario of false uniqueness (*χ* = −*δ* = −0.6). Finally, in a population with individuals that under-trust (*χ* = *δ* = −0.6), monetary incentives are ineffective to a large extent. Same parameters as Figure 3. Other parameters: *Z* = 100, *β* = 10, *μ* = 0.05. See also Figures S2–S5 for an extended exploration of incentives and biases in other games, as well as an exploration of different population sizes, group sizes, and selection intensities. See Figure S6 for heterogeneous, normally distributed biases in a population.

Establishing a quantitative link among incentives, bias, and collective success is only possible by considering the combined effect of the different equilibria and dynamical regions identified in Figures 3 and 4. So, now, we turn our attention to identifying the time that a population spends in each state and what the chance is that group success is achieved in those states. This can be accomplished by focusing on a finite population of size *Z* and analyzing the stochastic, individual decisions. We assume the same process as before but allow for a small probability of not adopting a strategy that is the best response (also called a smooth best response (Fudenberg et al., 1998), which mimics uncertainty in estimating the payoff differences of the order to 1/*β*) and randomly adopting any possible strategy (with probability *μ*). Moreover, we alter the game to include punishment applied to defectors (e.g., fines, higher tariffs, or taxes) by an amount *ιc*, 0≤*ι* ≤ 1. The value of *ι* represents how the fines imposed compare with the costs paid by cooperators, with *ι* = 0 meaning that no punishment is imposed and *ι* = 1 meaning that all the payoff advantage of defectors, when compared with cooperators, is removed. In Figure 6, we show that increasing the magnitude of punishment has a different effect depending on the nature of bias prevailing in a population. For instance, a lower punishment is necessary to sustain collective success under false consensus, compared with false uniqueness (for the combination of parameters analyzed, in particular, high value *M* = 8). In fact, the prevalent coexistence characterizing false uniqueness and identified in Figure 4A may lead to a fraction of cooperators that remains insufficient to guarantee high average levels of group success; circumventing such stable coexistence of cooperators and defectors proves to be harder—requiring extra incentives—than eliminating the neutral region associated with false consensus (Figure 4C). Additionally, we can observe that an effort to reduce individuals' perception biases can render high levels of collective success, even in situations where low incentives (low *ι*) are not effective—as a baseline, we show the group success characterizing a situation where neither biases nor incentives lead to the coordination in virtually all groups (Figure 6, gray curves).

Here, we assume that incentives are exogenously imposed (Góis et al., 2019) and do not introduce punishment strategies as in, e.g., Couto et al. (2020), Hauert et al. (2007), Quan et al. (2017), Roos et al. (2015), and Vasconcelos et al. (2013). Often, implementing incentives and institutions results in second-order free-riding dilemmas; we argue that, even if such dilemmas are solved, biases can affect the effectiveness of punishment and rewards. Also, we note that a direct comparison of the costs required to implement monetary-based incentives and information-based incentives is case sensitive, and future works can build on the model we propose for that purpose. Our results, however, already show that leveraging cooperation and group success may benefit from explicitly identifying and addressing individuals' social perception biases.

## Discussion

Understanding how cooperation can be sustained in public goods dilemmas of different kinds is central to address many of society's current challenges. That endeavor can benefit from recognizing the effect of social perception bias in cooperation dynamics and setting up incentives and interventions that understand and incorporate those dynamics. Here, we show that different types of social perception bias (e.g., false consensus, false uniqueness, over-trust, or under-trust) play a distinct role in the behavioral dynamics associated with non-linear public goods. Over-trust (under-trust) is likely to ease (hinder) the coordination associated with reaching the minimal number of contributors for cooperation to self-organize. False uniqueness leads to a persistent coexistence of cooperators and defectors, which can be insufficient to achieve collective success. Conversely, false consensus originates a neutral region where it is expected that individuals stick with their strategies, possibly changing behaviors only through exploration (Traulsen et al., 2009) and motives extraneous to the game being played. The fact that biases generate new, stable equilibria can have strong implications for the functioning of society. The workings and efficiency of markets and market regulation rely on the bottom-up ability of selfish agents to achieve socially desired outcomes and not get stuck in deadlocks as the ones we identify. Furthermore, these new equilibria are damaging for the possibility to coordinate from unfavorable into highly favorable states. They halt such a transition even in situations when all individuals would personally benefit from it. Besides implying different dynamics, such biases can render incentives less effective: as a prototypical example, false uniqueness requires that additional punishment is imposed on defectors (or, equivalently, rewards on cooperators) to achieve the same levels of group success, when compared with, e.g., populations under the effect of false consensus, and both require severer punishment compared with the absence of biases.

Although, currently, we focus on populations homogeneous in terms of bias and social contacts, the mathematical framework we propose can, in the future, be tuned to explicitly consider differences in biases within the same populations (Pearson et al., 2018) and the extent to which different network topologies may augment the effect of perception bias on cooperation. In fact, some authors suggest that social biases and judgment errors are often contradictory (Krueger and Funder, 2004). In this regard, considering the social network of interacting individuals not only may prove desirable to re-create realistic settings but also can be instrumental in explaining the origin of social perception biases and reconciling the apparently contradictory ones. Lee et al. show that considering homophily and interactions over a social network can help to explain seemingly conflicting biases, such as the overestimation and underestimation of a minority group size (Lee et al., 2019). Similarly, Galesic et al. show that homophily and a sampling process whereby individuals derive their judgments from local information based on their social environment (e.g., family, friends, and acquaintances) can explain when false consensus or false uniqueness is expected to occur (Galesic et al., 2018). Alipourfard et al. further show that individuals' perceptions can be biased as a result of local correlations in a directed social network (Alipourfard et al., 2020), and Lerman et al. show that social network effects can lead individuals to overestimate states that are globally rare, if those are overrepresented in their local neighborhoods—a phenomenon named majority illusion (Lerman et al., 2016). If perception biases result from social network effects rather than cognitive flaws, interventions based on reshaping information flows about global behaviors are possible and can be very impactful.

The analysis performed here is particularly relevant and timely given the growing number of works showing that individuals systematically under- or overestimate the position of others in matters affecting collective action problems (Kjeldahl and Hendricks, 2018; Leviston et al., 2013; Mildenberger and Tingley, 2019; Monin and Norton, 2003; Pearson et al., 2018) (also beyond climate change [Suls et al., 1988]). In fact, such perception biases are only but a subset of cognitive barriers that might affect decision making and impede collective action toward a better future (Weber, 2017). To reason about how those biases come to be and change over time is indispensable for a mechanistic understanding of the feedbacks between interventions and the biases themselves. As mentioned above, the existence of perception bias can be a by-product of individuals' psychological states, as well as the influence of local assortment (Cooney et al., 2016; Lee et al., 2019), specific network topologies (Alipourfard et al., 2020), or information filtering. False consensus, particularly, is likely to emerge if individuals' opinions assort them. Establishing a link between bias and cooperation can further illuminate how cooperation dynamics can depend on factors such as opinion polarization and assortment (McCarty, 2019), echo chambers (Colleoni et al., 2014), information cocoons (Sunstein, 2007), or on decisions about which opinions to share on mass media (Bowen et al., 2021; Boykoff and Boykoff, 2004; Feldman et al., 2012). To realize the emergence and persistence of these biases, one can also focus on the coevolutionary dynamics of strategic behavior at par with the evolutionary dynamics of beliefs (Galesic et al., 2021) and biases (Johnson and Fowler, 2011; Leimar and McNamara, 2019).

Different issues can also be associated with different levels of perception biases. Those levels depend on how visible issues are (Shamir and Shamir, 1997) and how visible the number of individuals supporting them is. Visibility can be a matter of design (e.g., using a COVID-19 tracing app entails the decision to give up privacy and contribute to a public good; informing how many people are using it is a decision of the designer) and policy-making (Nyborg et al., 2016). As Bicchieri puts it, solving *collective action traps* may require a *collective change of expectations* (Bicchieri, 2016). In this regard, our work provides a mechanistic understanding of how norm-based interventions (aiming at changing individuals' perceptions and expectations [Carattini et al., 2019; Miller and Prentice, 2016; Prentice and Paluck, 2020; Tankard and Paluck, 2016]) and information design (Mathevet et al., 2020) can be fundamental tools to trigger and sustain collective action.

### Limitations of the study

The current study focuses on dilemmas that consist of the binary decision to cooperate or defect. Furthermore, we do not model explicitly how perception biases evolve. Future studies can address these limitations by extending the proposed model to understand the role of perception biases in dilemmas with continuous contribution decisions (e.g., deciding how much to contribute to collective success from a range of possible contributions), strategies explicitly conditioned on the number of expected cooperators (Ohtsuki 2018) and in contexts where biases can evolve at par with strategies. Bias dynamics can be studied in several ways: On the one hand, as introduced earlier, different biases can emerge in particular network topologies and as a function of individuals' homophily degree (e.g., see Galesic et al., 2018; Lee et al., 2019; Lerman et al., 2016), which calls to consider biases and cooperation dynamics on top of interaction networks. On the other hand, the development of biases can be studied through evolutionary models that explicitly define perception biases' fitness (e.g., as in Johnson and Fowler [2011] where the evolution of overconfidence is studied in the context of conflicts over resources) or through multi-level selection models (Cooney, 2019), where groups with particular sizes, structures, and information dissemination tools can inspire or solve specific perception biases that affect internal cooperation levels and the consequent capacity to outperform other groups. Finally, here we assume that individuals can, at least, track the direction of shifts in cooperation levels correctly. One can argue that biases can also prevent detecting such changes. In this regard, we note that previous works establish a distinction between bias and accuracy (West and Kenny, 2011) such that individuals may systematically misperceive the real cooperation levels due to biases toward their own perspective but accurately track changes in cooperation over time. It would be relevant to investigate, in the future, how accurately perceiving changes can be instrumental in designing incentives for cooperation in the same dilemmas we here study.

### Resource availability

#### Lead contact

Further information and requests for materials should be directed to Fernando P. Santos (fppdsantos@gmail.com).

#### Materials availability

This study did not generate new unique reagents.

#### Data and code availability

The data that support the results of this study are available from the corresponding authors upon request. The figures discussed result directly from the set of equations described in the transparent methods section (Supplemental Information). The code used to implement such equations and generate the figures is available from the corresponding authors upon request.

## Methods

All methods can be found in the accompanying transparent methods supplemental file.

## Supplemental information

Document S1. Transparent methods and Figures S1–S6

## Footnotes

Supplemental information can be found online at https://doi.org/10.1016/j.isci.2021.102375.
