# Supplementary material for: Biased perceptions explain collective action deadlocks and suggest new mechanisms to prompt cooperation
Source: iScience. 2021 Mar 29;24(4):102375. doi: 10.1016/j.isci.2021.102375 (PMC8080528; doi:10.1016/j.isci.2021.102375)
Supplement: Document S1. Transparent methods and Figures S1–S6 [file mmc1.pdf]

iScience, Volume *24*

Supplemental information

Biased perceptions explain collective action deadlocks and suggest new mechanisms to prompt cooperation

Fernando P. Santos, Simon A. Levin, and Vítor V. Vasconcelos

Supplemental Figure 1

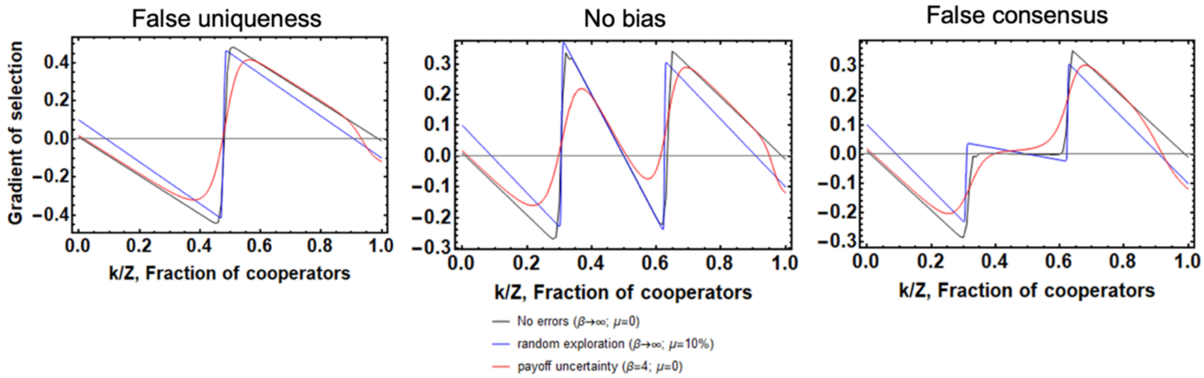

Figure S1. Effect of spontaneous changes (with probability μ, also known as mutation probability) and errors (controlled by the intensity of selection β, the largest β, the less errors individuals do when updating strategies) in the infinite population dynamics, Related to Figure 4. Other parameters: $\chi = \pm 0.2$, $\delta = \pm 0.2$, $f = 1.5$, $M = 8$.

Supplemental Figure 2

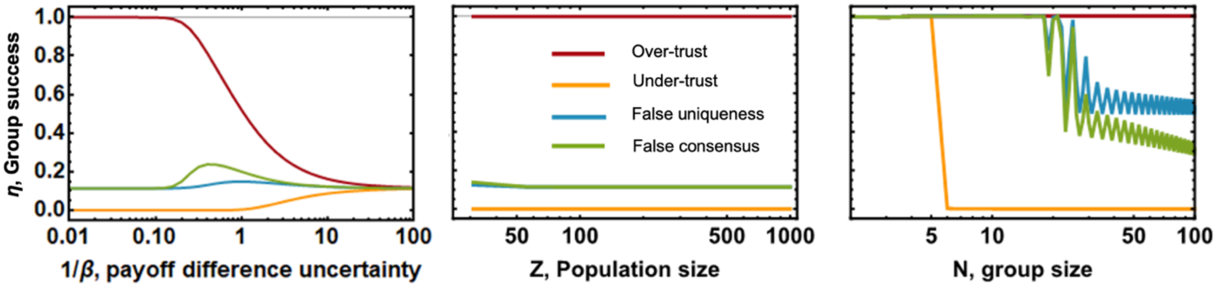

Figure S2. Role of payoff difference uncertainty (the inverse of selection intensity, $\mathbf{1/\beta}$), population size ($\boldsymbol{Z}$) and group size ($\boldsymbol{N}$) in overall group achievement, Related to Figure 6. When fixed, $Z = 100, M = 8, f = 1.5, N = 11, c = 1, b = 10, \beta = 10, \mu = 0.05$. $M =$ rounded$[0.5N]$ when N is varying.

Supplemental Figure 3

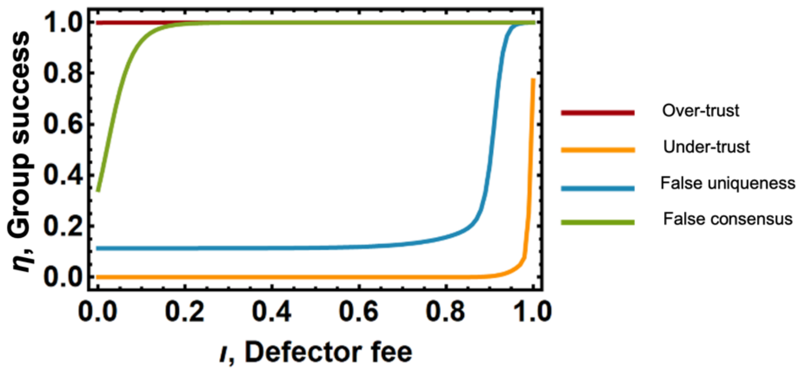

Figure S3. Effect of asymmetric biases, that is, deviation from diagonals in Figure 1, Related to Figure 6. Same parameters as Figure 6 in main text, but with $\chi = \pm 0.7, \delta = \pm 0.5$. Compare with Figure 6 of the main text.

Supplemental Figure 4

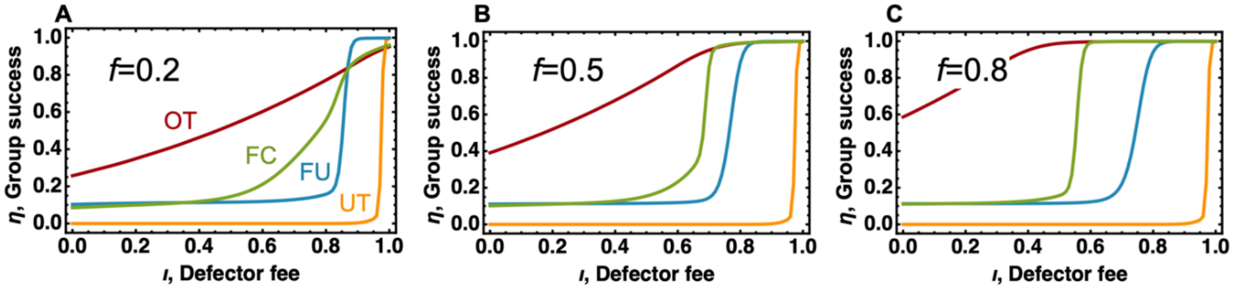

Figure S4. The role of incentives in populations with perception bias: Over-trust (OT), False consensus (FC), False uniqueness (FU) and Under-trust (UT), Related to Figure 6. Results for N-person game with co-existence (f<1), that is, where individuals do not have incentive to contribute further after the group has achieved the collective success threshold. Same parameters as Figure 6 in main text.

Supplemental Figure 5

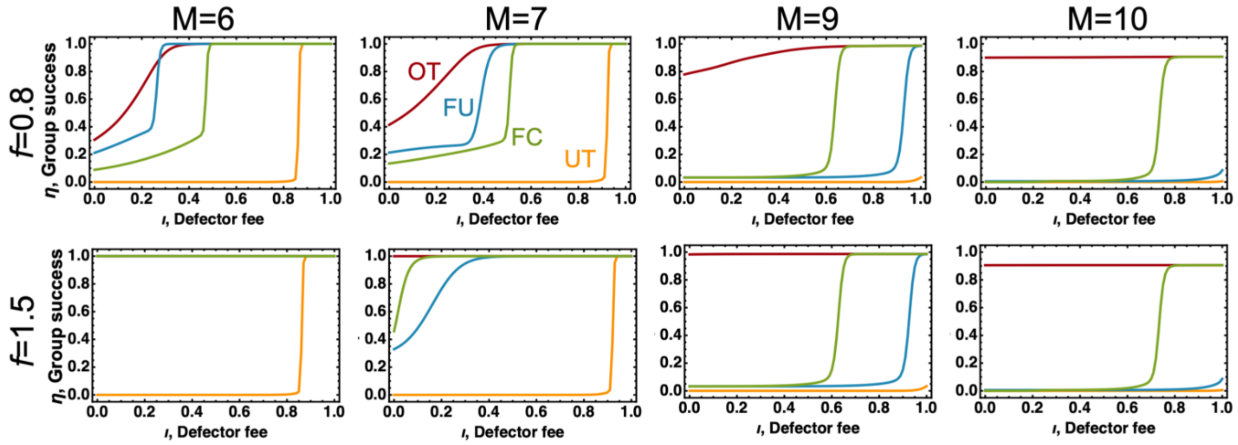

Figure S5. The role of incentives in populations with perception bias playing dillemas with different results from cooperation ($f = 0.8$ and $f = 1.5$) and group success threshold ($M = \{6, 7, 9, 10\}$), Related to Figure 6. Same parameters as Figure 6 in main text. We can observe that, as discussed in the main text, false consensus (FC) requires less punishment to achieve high values of collective success when M and *f* are both high. For low M and *f* we can also observe situations in which false uniqueness (FU) leads to scenarios where it becomes easier to incentivize cooperation. We also represent group success under over-trust (OT) and under-trust (UT).

Supplemental Figure 6

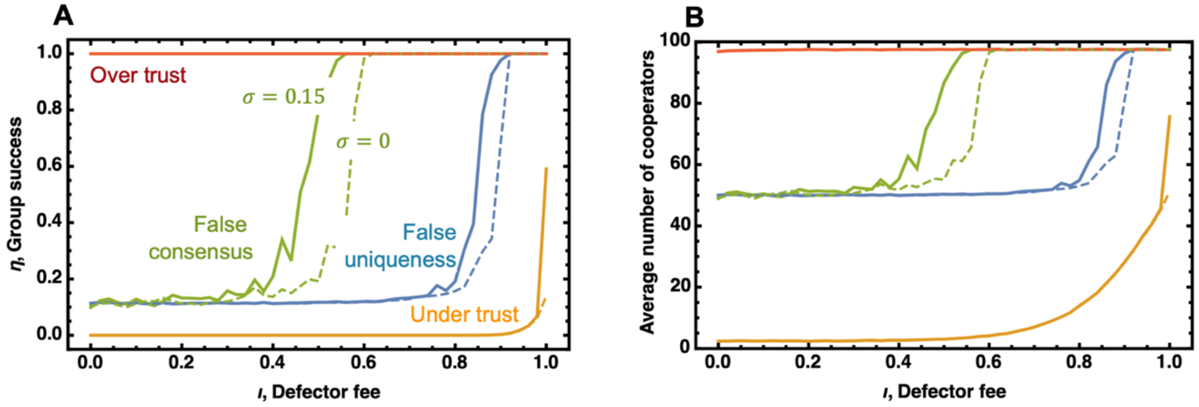

Figure S6. The role of incentives under bias heterogeneity, Related to Figure 6. Contrarily to the homogeneous bias scenario whose results we represent in Figure 6, here we assume that χ and δ convey the mean of a normal distribution with standard deviation σ. We compute, numerically, the average fraction of cooperators and average group success for each value of defector fee (ι), taken over 1000 generations (where, at each generation, Z individuals have the possibility of changing strategies). Instead of using the transition probabilities detailed in Equations (9) and (10) below — which preclude any difference among cooperators or among defectors — we explicitly sample individuals, each possibly characterized by a specific value of bias, and compute the probability that this unique individual changes strategy. Assuming that strategy updates follow this stochastic process (also accounting for a mutation probability, $\mu = 0.05$), we keep track of the number of strategies in the population in each generation, which allows us to quantify the average collective success (left panel) and average number of cooperators (right panel) associated with each value of fee ι. This way, we are able to compute the collective success assuming an arbitrary distribution of biases in the population. Each combination of cooperators' and defectors' biases (χ and δ) represents the mean of a Normal distribution with standard deviation σ. For each bias (false uniqueness, false consensus, over-trust, and under-trust), we use the same (χ,δ) as in Figure 6. We plot results for $\sigma = 0$ and $\sigma = 0.15$. Despite the noise associated with the numerical procedure we use here — note that now, on top of bias heterogeneity, to compute a smooth transition probability between states one needs a very large number of samples — we are able to confirm the results of Figure 6 in a scenario of bias heterogeneity. Same parameters as Figure 6. Dashed lines correspond to $\sigma = 0$ (homogeneous bias) and full lines to $\sigma = 0.15$ (heterogeneous bias).

Transparent Methods

Payoff: Players interact in groups of fixed size N to obtain a payoff Π_X that depends on their action, $X = C$ or D, and other players' actions. Action C corresponds to costly cooperation, and action D corresponds to defection. In an interacting group, cooperation costs an amount c, and, if there are less than M cooperators, there is no benefit to any of the group members. Whenever the group reaches a threshold of M cooperators, each individual gets a benefit, bc, plus an additional reward per extra cooperator, fc. Thus, if we let j be the number of cooperators in a group, we can write

$$\Pi_D[j] = \big(bc + fc(j - M)\big)\Theta[j - M] \text{ and} \tag{1}$$

$$\Pi_C[j] = \Pi_D[j] - c, \tag{2}$$

where $\Theta[x]$ is the unit step function, which is 0 for $x < 0$ and 1 for $x \geq 0$. In Figure 6, we alter the game to include punishment applied to defectors (e.g., fines, higher tariffs, or taxes), by an amount ιc, $0 \leq \iota \leq 1$. The value of ι represents how the fines imposed compare with the costs paid by cooperators, with $\iota = 0$ meaning that no punishment is imposed and $\iota = 1$ that all the payoff advantage of defectors, in comparison with cooperators, is removed. Eq. (1) is thereby modified to $\Pi_D[j] = \big(bc + fc(j - M)\big)\Theta[j - M] - \iota c$.

Infinite populations: At each time unit, individuals have the same probability of considering changing their strategy. Changing strategy depends on the outcome they expect to get from their interactions, given the number of cooperators (and defectors) they perceive will be present. An actor playing X will compare the expected payoff of cooperation, $f_C[\tilde{x}^X]$, to the average payoff of defectors, $f_D[\tilde{x}^X]$, when interacting in a group of size N, depending on the perceived fraction of cooperators in the population perceived by that player, $\tilde{x}^X$. Each individual assumes they are equally likely to interact with all others, resulting in expected payoffs of

$$f_C[\tilde{x}^X] = \sum_{k=0}^{N-1} \binom{N-1}{k} (\tilde{x}^X)^k (1 - \tilde{x}^X)^{N-1-k} \Pi_C[k+1] \text{ and} \tag{3}$$

$$f_D[\tilde{x}^X] = \sum_{k=0}^{N-1} \binom{N-1}{k} (\tilde{x}^X)^k (1 - \tilde{x}^X)^{N-1-k} \Pi_D[k]\,. \tag{4}$$

The player with strategy X will change to the strategy Y if the expected average payoff is not worse, with a probability of $p_{X\to Y}[f_Y - f_X] = \Theta[f_Y - f_X]$. Finally, the perceived fraction of cooperators by each strategy, though influenced by the actual number of cooperators in the population, x, is affected by biases, which only act on the strategies of the other players. If χ and δ represent the biases affecting cooperators and defectors, respectively, then a cooperator will estimate a fraction of cooperators $\tilde{x}^C[x] = x^{10^{-\chi}} = \exp\big[10^{-\chi}\ln[x]\big]$ and a defector will estimate a fraction of defectors $\tilde{x}^D[x] = x^{10^{-\delta}} = \exp\left[10^{-\delta}\ln[x]\right]$ (where the second equality serves to clarify that $-\chi$ and $-\delta$ are exponents of 10). The previous equalities are also usefull to clarify that the choice of basis 10 is arbitrary and does not affect the generality of our results; different basis can be considered and, by rescaling χ and δ, the same results would follow — for example, we could consider basis e instead of 10, $\tilde{x}^D[x] = x^{e^{-\bar{\delta}}}$ and $\tilde{x}^C[x] = x^{e^{-\bar{\chi}}}$, in which case the results under basis 10 are recovered by equating $\bar{\chi} = \ln[10]\chi$ and $\bar{\delta} = \ln[10]\delta$. This formulation guarantees that positive (negative) values of χ and δ indicate overestimation (underestimation) of cooperation (see Figure 1 in the main text). If $\chi = \delta = 0$ then $\tilde{x}^C[x] = \tilde{x}^D[x] = x$, which recovers the typical no-bias scenario where perceptions match reality. Thus, we can write the probability that the number of Cs increases, and the

number of Ds decreases, per time unit as $T^{+}[x] = (1-x)p_{D\to C}\left[f_C\left[\tilde{x}^D[x]\right] - f_D\left[\tilde{x}^D[x]\right]\right]$ and the probability that the number of Cs decreases, and the number of Ds increases, per time unit as $T^{-}[x] = x\, p_{C\to D}\left[f_D\left[\tilde{x}^C[x]\right] - f_C\left[\tilde{x}^C[x]\right]\right]$. The gradient of selection (Figure 4 and Figure S1) indicates the most likely direction of evolution of the population and is given by $g[x] = T^{+}[x] - T^{-}[x]$; when $g[x] > 0$, the number of cooperators is likely to increase and $g[x] < 0$ implies that cooperation is likely to decrease.

Finite populations: Let us now consider a population of size Z. As before, players interact in groups of fixed size $N \leq Z$ to obtain a payoff Π_X that depends on their action, $X = C$ or D, and other players' actions (as detailed above).

Each time unit, individuals have the same probability of considering changing their strategy based on the outcome they expect to get from their interactions, given the number of cooperators (and defectors) they perceive will be present. An actor playing X will compare the average payoff of cooperation, $f_C[\tilde{x}^X]$, to the average payoff of defectors, $f_D[\tilde{x}^X]$, depending on the perceived fraction of cooperators in the population seen by that player, $\tilde{x}^X$. We assume a complete graph of interactions from which the interaction groups are sampled, resulting in average payoffs of

$$f_C[\tilde{x}^X] = \sum_{k=0}^{N-1} \binom{Z-1}{N-1}^{-1} \binom{Z\,\tilde{x}^X - 1}{k} \binom{Z(1-\tilde{x}^X)}{N-1-k} \Pi_C[k+1] \text{ and} \tag{5}$$

$$f_D[\tilde{x}^X] = \sum_{k=0}^{N-1} \binom{Z-1}{N-1}^{-1} \binom{Z\,\tilde{x}^X}{k} \binom{Z(1-\tilde{x}^X)-1}{N-1-k} \Pi_D[k]\,. \tag{6}$$

The player with strategy X will change to strategy Y with a probability that increases with the difference of expected average payoffs. The player can also change spontaneously from one strategy to another at a rate μ, due to some exogenous event. Combining those effects resuls in a probability of changing strategy of $\mu + (1-\mu)\left(1 + e^{-\beta(f_Y - f_X)}\right)^{-1}$. Finally, the actual fraction of cooperators in the population, x, affects the perceived fraction of cooperators by each strategy. However, biases on the strategies of others also affect the latter. If χ and δ represent the bias affecting cooperators and defectors, respectively, then a cooperator will estimate a fraction of cooperators $\tilde{x}^C[x]$ and a defector will estimate a fraction of defectors $\tilde{x}^D[x]$ given by

$$\tilde{x}^C[x] = \left(\frac{Zx-1}{Z-1}\right)^{10^{-\chi}} + \frac{1}{Z} \text{ and} \tag{7}$$

$$\tilde{x}^D[x] = \left(\frac{Zx}{Z-1}\right)^{10^{-\delta}}\,. \tag{8}$$

This formulation guarantees that positive (negative) values of χ and δ indicate overestimation (underestimation) of cooperation. Again, we note that, in Eqs. (7) and (8), $-\chi$ and $-\delta$ are exponents of 10, and basis 10 was chosen without loss of generality. Thus, we can write the probability that the number of Cs increases, and the number of Ds decreases, per time unit, $T^{+}[x]$, and the probability that the number of Cs decreases, and the number of Ds increases, per time unit, $T^{-}[x]$, as

$$T^{+}[x] = (1-x)\left(\mu + (1-\mu)\left(1 + e^{-\beta\left(f_C\left[\tilde{x}^D[x] + \frac{1}{Z}\right] - f_D\left[\tilde{x}^D[x]\right]\right)}\right)^{-1}\right) \text{ and} \tag{9}$$

$$T^{-}[x] = x\left(\mu + (1-\mu)\left(1 + e^{-\beta\left(f_D\left[\tilde{x}^C[x] - \frac{1}{Z}\right] - f_C\left[\tilde{x}^C[x]\right]\right)}\right)^{-1}\right). \tag{10}$$

We note that this update resembles a smooth best-response (Fudenberg et al., 1998) and, in the past, was also used to model so-called counterfactual thinking (Pereira and Santos, 2018). As when considering infinite populations, the gradient of selection indicates the most likely direction of evolution of the population and is, thus, given by $g[x] = T^{+}[x] - T^{-}[x]$. In this case, diffusion indicates the level of noise of the system at any configuration and is given by $d[x] = (T^{+}[x] + T^{-}[x])/Z$.

Analysis of the dynamics for infinite populations

The gradient of selection and diffusion govern the dynamics, which can be written as:

$$\dot{x} = g[x] + \sqrt{d[x]}\,\Gamma[t], \tag{11}$$

where $\Gamma[t]$ is a random variable with gaussian distribution of zero mean and unit variance. Thus, when $g[x]$ is positive, x tends to increase. When $g[x]$ is negative, x tens to decrease. The sign of g alone contains the information of the preferential direction of evolution of the fraction of cooperators in the population.

In the case of an infinite population, $Z \to \infty$, and perfect best response, $\beta \to \infty$, we get

$$\dot{x} = \mu(1-2x) + (1-\mu)\left((1-x)\,\Theta\left[f_C\left[\tilde{x}^D[x]\right] - f_D\left[\tilde{x}^D[x]\right]\right] - x\,\Theta\left[f_D\left[\tilde{x}^C[x]\right] - f_C\left[\tilde{x}^C[x]\right]\right]\right), \tag{12}$$

and

$$f_C[\tilde{x}^X] = \sum_{k=\mathrm{M}-1}^{N-1} \binom{N-1}{k} (\tilde{x}^X)^k (1-\tilde{x}^X)^{N-1-k} (bc + fc(k-M) + fc) - c \tag{13}$$

$$f_D[\tilde{x}^X] = \sum_{k=\mathrm{M}}^{N-1} \binom{N-1}{k} (\tilde{x}^X)^k (1-\tilde{x}^X)^{N-1-k} \big(bc + fc(k-M)\big) \tag{14}$$

and

$$\begin{aligned}
f_C[\tilde{x}^X] - f_D[\tilde{x}^X] &= \sum_{k=\mathrm{M}-1}^{N-1} \binom{N-1}{k} (\tilde{x}^X)^k (1-\tilde{x}^X)^{N-1-k} (bc + fc(k-M) + fc) - c \\
&\quad - \sum_{k=\mathrm{M}}^{N-1} \binom{N-1}{k} (\tilde{x}^X)^k (1-\tilde{x}^X)^{N-1-k} \big(bc + fc(k-M)\big) \\
&= bc \binom{N-1}{\mathrm{M}-1} (\tilde{x}^X)^{\mathrm{M}-1} (1-\tilde{x}^X)^{N-M} + fc \sum_{k=\mathrm{M}}^{N-1} \binom{N-1}{k} (\tilde{x}^X)^k (1-\tilde{x}^X)^{N-1-k} - c \\
&= fc(1 - \mathrm{CDF}[\mathrm{Binomial}[N-1, \tilde{x}^X], M-1]) + \binom{N-1}{M} (\tilde{x}^X)^{M-1} (1-\tilde{x}^X)^{N-M} bc - c.
\end{aligned}$$

Notice that $f_C[0] - f_D[0] = -c < 0$ and, when $f > 1$, $f_C[1] - f_D[1] = fc - c > 0$. If $X = D$, this change of signs guarantees that, from the perspective of a defector, there is at least one coordination dilemma, i.e., there is no incentive to change strategy if there are too few cooperators, and there is an incentive to become a cooperator if there are enough cooperators. Identically for the perspective of cooperators, when $X = C$, irrespectively of the bias function.

Analysis of the dynamics for finite populations

Recovering Eqs.(5-6) and Eqs.(1-2), we can write

$$
\begin{aligned}
f_D[\tilde{x}^X] &= \sum_{k=0}^{N-1} \binom{Z-1}{N-1}^{-1} \binom{Z\,\tilde{x}^X}{k} \binom{Z(1-\tilde{x}^X)-1}{N-1-k} \Pi_D[k] \\
&= \sum_{k=0}^{N-1} \binom{Z-1}{N-1}^{-1} \binom{Z\,\tilde{x}^X}{k} \binom{Z(1-\tilde{x}^X)-1}{N-1-k} \big(bc + fc(k-M)\big)\Theta[k-M] \\
&= \sum_{k=M}^{N-1} \binom{Z-1}{N-1}^{-1} \binom{Z\,\tilde{x}^X}{k} \binom{Z(1-\tilde{x}^X)-1}{N-1-k} \big(bc + fc(k-M)\big)
\end{aligned}
\tag{15}
$$

$$
\begin{aligned}
f_C[\tilde{x}^X] &= \sum_{k=0}^{N-1} \binom{Z-1}{N-1}^{-1} \binom{Z\,\tilde{x}^X - 1}{k} \binom{Z(1-\tilde{x}^X)-\delta_{XD}}{N-1-k} \Pi_C[k+1] \\
&= \sum_{k=0}^{N-1} \binom{Z-1}{N-1}^{-1} \binom{Z\,\tilde{x}^X - 1}{k} \binom{Z(1-\tilde{x}^X)}{N-1-k} \big(bc + fc(k+1-M)\big)\Theta[k+1-M] - c \\
&= \sum_{k=\mathrm{M}-1}^{N-1} \binom{Z-1}{N-1}^{-1} \binom{Z\,\tilde{x}^X - 1}{k} \binom{Z(1-\tilde{x}^X)}{N-1-k} \big(bc + fc(k+1-M)\big) - c \\
&= \binom{Z-1}{N-1}^{-1} \binom{Z\,\tilde{x}^X - 1}{M-1} \binom{Z(1-\tilde{x}^X)}{N-\mathrm{M}} bc + \\
&\quad + \sum_{k=\mathrm{M}}^{N-1} \binom{Z-1}{N-1}^{-1} \binom{Z\,\tilde{x}^X - 1}{k} \binom{Z(1-\tilde{x}^X)}{N-1-k} \big(bc + fc(k+1-M)\big) - c
\end{aligned}
\tag{16}
$$

To compute T^+ we need

$$
\begin{aligned}
&f_C\left[\tilde{x}^D[x] + \frac{1}{Z}\right] - f_D\big[\tilde{x}^D[x]\big] = \\
&\qquad = \binom{Z-1}{N-1}^{-1} \binom{Z\,\tilde{x}^D}{M-1} \binom{Z(1-\tilde{x}^D)-1}{N-\mathrm{M}} bc + \\
&\qquad\quad + fc \sum_{k=\mathrm{M}}^{N-1} \binom{Z-1}{N-1}^{-1} \binom{Z\,\tilde{x}^D}{k} \binom{Z(1-\tilde{x}^D)-1}{N-1-k} - c \\
&\qquad = fc\big(1 - \mathrm{CDF}\big[\mathrm{HyperGeo}[Z-1, N-1, Z\tilde{x}^D], M-1\big]\big) + \\
&\qquad\quad + \binom{Z-1}{N-1}^{-1} \binom{Z\,\tilde{x}^D}{M-1} \binom{Z(1-\tilde{x}^D)-1}{N-\mathrm{M}} bc - c.
\end{aligned}
\tag{17}
$$

To compute T^- we need

$$
\begin{aligned}
&f_D\left[\tilde{x}^C[x] - \frac{1}{Z}\right] - f_C\big[\tilde{x}^C[x]\big] = \\
&\qquad = -\binom{Z-1}{N-1}^{-1} \binom{Z\,\tilde{x}^C - 1}{M-1} \binom{Z(1-\tilde{x}^C)}{N-M} bc - \\
&\qquad\quad - fc \sum_{k=\mathrm{M}}^{N-1} \binom{Z-1}{N-1}^{-1} \binom{Z\,\tilde{x}^C - 1}{k} \binom{Z(1-\tilde{x}^C)}{N-1-k} + c \\
&\qquad = -fc\big(1 - \mathrm{CDF}\big[\mathrm{HyperGeo}[Z-1, N-1, Z\tilde{x}^C - 1], M-1\big]\big) - \\
&\qquad\quad - \binom{Z-1}{N-1}^{-1} \binom{Z\,\tilde{x}^C - 1}{M-1} \binom{Z(1-\tilde{x}^C)}{N-M} bc + c.
\end{aligned}
\tag{18}
$$

Importantly, we can write

$$\tilde{x}^X[x] = \left(\frac{Zx - \delta_{XC}}{Z-1}\right)^{10^{-b_X}} + \frac{\delta_{XC}}{Z}, \quad (19)$$

with $b_X = \chi\delta_{XC} + \delta\, \delta_{XD}$. Here, δ_{XY} represents the Kroneker delta and should not be confused with δ (without subscripts and representing defectors' bias): $\delta_{XY} = 1$ if $X = Y$ and $\delta_{XY} = 0$ otherwise. For any $\mu > 0$ and finite β we can define a Markov chain of the number of cooperators over time, i, using the probabilities of increasing and decreasing i by one unit as $T^+[i/Z]$ and $T^-[i/Z]$, respectively. The evolution of i is governed by a Master-equation of the form

$$\frac{dp_i[t]}{dt} = p_{i-1}[t]T^+\left[\frac{i-1}{Z}\right] + p_{i+1}[t]T^-\left[\frac{i+1}{Z}\right] - p_i[t]\left(T^+\left[\frac{i}{Z}\right] + T^-\left[\frac{i}{Z}\right]\right), \quad (20)$$

where $p_i[t]$ is the probability of finding the system in configuration i after a period t in which the system was in some configuration i_0, $p_i[0] = \delta_{ii_0}$. The solution will converge to a stationary solution, p_i^*, which is independent of the initial condition i_0. Thus, p_i^* reflects the probability of finding the system with i cooperators a longer time after we observe i_0 cooperators (which is our best bet if there are no observations at all).

With it, we can compute the expected number of groups that reach the threshold, which we call group achievement, η. The group achievement is computed as

$$\eta = \sum_{i=0}^{Z} p_i^* \sum_{k=M}^{N} \binom{Z}{N}^{-1} \binom{i}{k} \binom{Z-i}{N-k}. \quad (21)$$

We can also compute the average level of cooperation simply as

$$\langle \frac{i}{Z} \rangle = \sum_{i=0}^{Z} p_i^* \frac{i}{Z}. \quad (22)$$

A note on the definition of false uniqueness

We note that the operationalization of false uniqueness that we use throughout our main text does not perfectly match all previous definitions of this social perception bias: false uniqueness was referred to as the tendency for individuals to underestimate the proportion of those sharing their desirable attributes (Baumeister and Vohs, 2007; Suls et al., 1988); an alternative would be using pluralistic ignorance, previously defined as the tendency for individuals to wrongly assume that their behaviors differ from everybody else's, which happens as public actions can differ from private beliefs and opinions (Baumeister and Vohs, 2007; Miller and McFarland, 1987). A completely accurate implementation of false uniqueness would require defining desirability, while a completely accurate implementation of pluralistic ignorance would require distinguishing public and private strategies in or model. As explicitly introducing desirability or private behaviors would increase the complexity of our model beyond the scope of the analysis we intend to perform (for the complexity of modeling desirability and private information associated with cooperation see, respectively, (Ohtsuki and Iwasa, 2004; Santos et al., 2018) and (Hilbe et al., 2018; Ohtsuki et al., 2015)), we opted to use false uniqueness to simply denote the tendency for individuals to underestimate the representativeness of their own strategy in the population, following works such as (Galesic et al., 2018; Krueger, 2000; Lee et al., 2019).

Supplemental References

Baumeister, R.F., Vohs, K.D., 2007. Encyclopedia of social psychology. Sage.

Fudenberg, D., Drew, F., Levine, D.K., Levine, D.K., 1998. The theory of learning in games. MIT press.

Galesic, M., Olsson, H., Rieskamp, J., 2018. A sampling model of social judgment. Psychological review 125, 363.

Hilbe, C., Schmid, L., Tkadlec, J., Chatterjee, K., Nowak, M.A., 2018. Indirect reciprocity with private, noisy, and incomplete information. Proceedings of the National Academy of Sciences 115, 12241–12246.

Krueger, J., 2000. The projective perception of the social world, in: Handbook of Social Comparison. Springer, pp. 323–351.

Lee, E., Karimi, F., Wagner, C., Jo, H.-H., Strohmaier, M., Galesic, M., 2019. Homophily and minority-group size explain perception biases in social networks. Nature Human Behaviour 3, 1078–1087.

Miller, D.T., McFarland, C., 1987. Pluralistic ignorance: When similarity is interpreted as dissimilarity. Journal of Personality and social Psychology 53, 298.

Ohtsuki, H., Iwasa, Y., 2004. How should we define goodness?—reputation dynamics in indirect reciprocity. Journal of theoretical biology 231, 107–120.

Ohtsuki, H., Iwasa, Y., Nowak, M.A., 2015. Reputation effects in public and private interactions. PLoS Comput Biol 11, e1004527.

Pereira, L.M., Santos, F.C., 2018. Counterfactual thinking in cooperation dynamics. Presented at the International conference on Model-Based Reasoning, Springer, pp. 69–82.

Santos, F.P., Santos, F.C., Pacheco, J.M., 2018. Social norm complexity and past reputations in the evolution of cooperation. Nature 555, 242–245.

Suls, J., Wan, C.K., Sanders, G.S., 1988. False consensus and false uniqueness in estimating the prevalence of health-protective behaviors. Journal of Applied Social Psychology 18, 66–79.
